# Supplementary material for: Differences in walking access to healthcare facilities between formal and informal areas in 19 sub-Saharan African cities
Source: Commun Med (Lond). 2025 Feb 14;5:41. doi: 10.1038/s43856-025-00746-5 (PMC11828986; doi:10.1038/s43856-025-00746-5)
Supplement: Supplementary file 2 — Supplementary Information [file 43856_2025_746_MOESM2_ESM.pdf]

Supplementary Information for Differences in walking access to healthcare facilities between formal and informal areas in 19 sub-Saharan African cities

John Friesen, Stefanos Georganos, Jan Haas

January 2025

Table S1: Relative risk of individuals living in informal areas of having a worse SA to hospitals (Group (i)) than individuals in formal areas

City	Relative Risk 60 min	Relative Risk 120 min
Accra	0.72 [0.45 - 1.24]	1.20 [0.91 - 1.49]
Addis Ababa	**1.46 [1.17 - 1.79]**	**1.22 [1.12 - 1.49]**
Arusha	1.14 [0.86 - 1.53]	**1.04 [1.00 - 1.20]**
Bamako	0.95 [0.72 - 1.24]	**1.15 [1.06 - 1.25]**
Beira	0.83 [0.43 - 1.59]	**1.48 [1.24 - 1.82]**
Gombe	1.53 [0.78 - 2.39]	1.07 [0.79 - 1.50]
Ibadan	0.79 [0.57 - 1.14]	1.05 [0.90 - 1.19]
Johannesburg	0.71 [0.31 - 1.75]	1.24 [0.81 - 1.91]
Kampala	0.63 [0.41 - 1.00]	0.94 [0.85 - 1.04]
Khartoum	**1.44 [1.13 - 1.88]**	**1.20 [1.11 - 1.41]**
Kigali	0.94 [0.57 - 1.40]	1.04 [0.98 - 1.19]
Kinsasha	0.29 [0.14 - 0.52]	0.94 [0.58 - 1.14]
Lagos	0.44 [0.30 - 0.71]	0.78 [0.58 - 1.03]
Luanda	0.94 [0.65 - 1.50]	1.05 [0.85 - 1.36]
Lubumbashi	2.03 [0.92 - 4.25]	**1.73 [1.09 - 3.00]**
Nakuru	0.43 [0.10 - 0.98]	1.10 [0.87 - 1.27]
Oyo	0.64 [0.46 - 1.05]	**1.03 [1.02 - 1.06]**
Port-Elizabeth	1.33 [0.67 - 2.34]	**1.66 [1.31 - 2.14]**

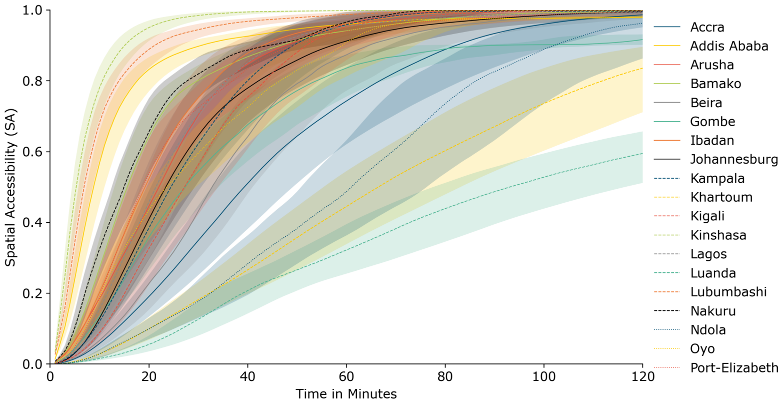

Figure S1: Spatial accessibility to all healthcare facilities (hospitals, clinics and smaller health facilities - Group (ii)) for full urban areas with uncertainty.

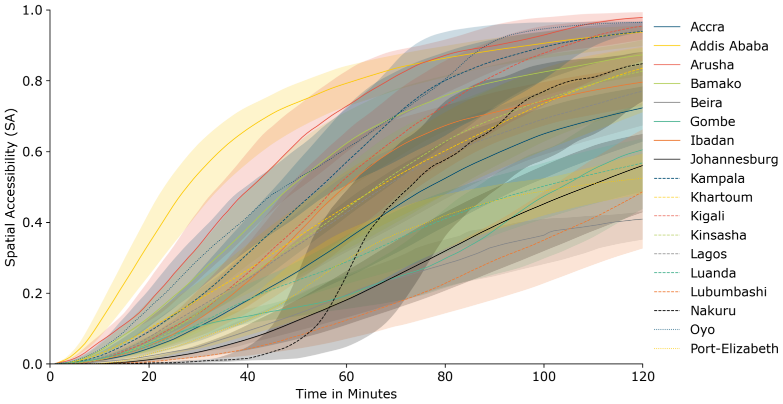

Figure S2: Spatial accessibility to hospitals (Group (i)) within 120 min.
