# Supplementary material for: Differences in walking access to healthcare facilities between formal and informal areas in 19 sub-Saharan African cities
Source: Commun Med (Lond). 2025 Feb 14;5:41. doi: 10.1038/s43856-025-00746-5 (PMC11828986; doi:10.1038/s43856-025-00746-5)
Supplement: Supplementary file 3 — Description of Additional Supplementary Files [file 43856_2025_746_MOESM3_ESM.pdf]

Description of Additional Supplementary Files

File name- Supplementary Data 1

File description- Source Data for Figure 1.

File name- Supplementary Data 2

File description- Source Data for Figure 2.

File name- Supplementary Data 3

File description- Source Data for Figure 3.

File name- Supplementary Data 4

File description- Source Data for Figure 4.

File name- Supplementary Data 5

File description- Classification of Healthcare facilities.
