# Supplementary material for: Differences in walking access to healthcare facilities between formal and informal areas in 19 sub-Saharan African cities
Source: Commun Med (Lond). 2025 Feb 14;5:41. doi: 10.1038/s43856-025-00746-5 (PMC11828986; doi:10.1038/s43856-025-00746-5)
Supplement: Supplementary file 9 — Reporting Summary [file 43856_2025_746_MOESM9_ESM.pdf]

nature portfolio

Corresponding author(s): John Friesen

Last updated by author(s): Nov 7, 2024

Reporting Summary

Nature Portfolio wishes to improve the reproducibility of the work that we publish. This form provides structure for consistency and transparency in reporting. For further information on Nature Portfolio policies, see our Editorial Policies and the Editorial Policy Checklist.

Statistics

For all statistical analyses, confirm that the following items are present in the figure legend, table legend, main text, or Methods section.

n/a	Confirmed	
☒	☐	The exact sample size (*n*) for each experimental group/condition, given as a discrete number and unit of measurement
☒	☐	A statement on whether measurements were taken from distinct samples or whether the same sample was measured repeatedly
☒	☐	The statistical test(s) used AND whether they are one- or two-sided<br>*Only common tests should be described solely by name; describe more complex techniques in the Methods section.*
☒	☐	A description of all covariates tested
☒	☐	A description of any assumptions or corrections, such as tests of normality and adjustment for multiple comparisons
☐	☒	A full description of the statistical parameters including central tendency (e.g. means) or other basic estimates (e.g. regression coefficient) AND variation (e.g. standard deviation) or associated estimates of uncertainty (e.g. confidence intervals)
☒	☐	For null hypothesis testing, the test statistic (e.g. *F*, *t*, *r*) with confidence intervals, effect sizes, degrees of freedom and *P* value noted<br>*Give P values as exact values whenever suitable.*
☒	☐	For Bayesian analysis, information on the choice of priors and Markov chain Monte Carlo settings
☒	☐	For hierarchical and complex designs, identification of the appropriate level for tests and full reporting of outcomes
☒	☐	Estimates of effect sizes (e.g. Cohen's *d*, Pearson's *r*), indicating how they were calculated

Our web collection on statistics for biologists contains articles on many of the points above.

Software and code

Policy information about availability of computer code

Data collection	We used four different datasets to conduct our analysis: WorldPop data for the 19 cities analysed (constrained UN adjusted data set for the year 2020 - downloaded in September 2023); OSM data on Street networks in the analysed cities downloaded in September 2023; data on landuse in the analysed cities (https://code.earthengine.google.com/?asset=projects/wri-datalab/urban_land_use/V1) downloaded in September 2023; data on healthcare facilities in Sub Saharan Africa (https://www.nature.com/articles/s41597-019-0142-2) downloaded in September 2023.
Data analysis	As described in the paper we used ArcGIS Pro 3.2.1 and the Network Analyst extension to calculate the service areas around the hospitals. We exported the shapefiles for the different travel times. We used the code from Breuer and Friesen to merge the WorldPop data with these shapefiles (https://doi.org/10.48328/tudatalib-1122.2). The resulting information on population per service area is analysed with the code provided in the code, provided in the paper (https://github.com/johnfriesen/Accessibility-Sub-Saharan-Africa).

For manuscripts utilizing custom algorithms or software that are central to the research but not yet described in published literature, software must be made available to editors and reviewers. We strongly encourage code deposition in a community repository (e.g. GitHub). See the Nature Portfolio guidelines for submitting code & software for further information.

Data

Policy information about availability of data

All manuscripts must include a data availability statement. This statement should provide the following information, where applicable:

- Accession codes, unique identifiers, or web links for publicly available datasets
- A description of any restrictions on data availability
- For clinical datasets or third party data, please ensure that the statement adheres to our policy

The gridded population data from WorldPop is available under https://hub.worldpop.org/geodata/listing?id=79. The data on healthcare facilities is described in the following publication: https://www.nature.com/articles/s41597-019-0142-2. The data on landuse in the analysed cities is available under https://code.earthengine.google.com/?asset=projects/wri-datalab/urban_land_use/V1.

Human research participants

Policy information about studies involving human research participants and Sex and Gender in Research.

Reporting on sex and gender	N/A
Population characteristics	N/A
Recruitment	N/A
Ethics oversight	N/A

Note that full information on the approval of the study protocol must also be provided in the manuscript.

Field-specific reporting

Please select the one below that is the best fit for your research. If you are not sure, read the appropriate sections before making your selection.

☐ Life sciences ☒ Behavioural & social sciences ☐ Ecological, evolutionary & environmental sciences

For a reference copy of the document with all sections, see nature.com/documents/nr-reporting-summary-flat.pdf

Behavioural & social sciences study design

All studies must disclose on these points even when the disclosure is negative.

Study description	We performed an analysis on spatial (physical) accessibliity to healthcar facilties in 19 African cities.
Research sample	We used the most comprehensive dataset on landuse available, covering not only the major, but also secondary cities.
Sampling strategy	N/A
Data collection	N/A
Timing	N/A
Data exclusions	N/A
Non-participation	N/A
Randomization	N/A

Reporting for specific materials, systems and methods

We require information from authors about some types of materials, experimental systems and methods used in many studies. Here, indicate whether each material, system or method listed is relevant to your study. If you are not sure if a list item applies to your research, read the appropriate section before selecting a response.

Materials & experimental systems

n/a	Involved in the study
☒	☐ Antibodies
☒	☐ Eukaryotic cell lines
☒	☐ Palaeontology and archaeology
☒	☐ Animals and other organisms
☒	☐ Clinical data
☒	☐ Dual use research of concern

Methods

n/a	Involved in the study
☒	☐ ChIP-seq
☒	☐ Flow cytometry
☒	☐ MRI-based neuroimaging
